# Correction: Dominant-negative mutations in human *IL6ST* underlie hyper-IgE syndrome

**DOI:** 10.1084/jem.2019180405272020c

**Published:** 2020-06-09

**Authors:** Vivien Béziat, Simon J. Tavernier, Yin-Huai Chen, Cindy S. Ma, Marie Materna, Arian Laurence, Jens Staal, Dominik Aschenbrenner, Lisa Roels, Lisa Worley, Kathleen Claes, Lisa Gartner, Lisa A. Kohn, Marieke De Bruyne, Klaus Schmitz-Abe, Louis-Marie Charbonnier, Sevgi Keles, Justine Nammour, Natasha Vladikine, Majistor Raj Luxman Maglorius Renkilaraj, Yoann Seeleuthner, Mélanie Migaud, Jérémie Rosain, Mohamed Jeljeli, Bertrand Boisson, Eva Van Braeckel, Jill A. Rosenfeld, Hongzheng Dai, Lindsay C. Burrage, David R. Murdock, Bart N. Lambrecht, Véronique Avettand-Fenoel, Tiphanie P. Vogel, Undiagnosed Diseases Network, Charles R. Esther, Sule Haskologlu, Figen Dogu, Peter Ciznar, David Boutboul, Marie Ouachée-Chardin, Jean Amourette, Marie-Noëlle Lebras, Clément Gauvain, Colas Tcherakian, Aydan Ikinciogullari, Rudi Beyaert, Laurent Abel, Joshua D. Milner, Bodo Grimbacher, Louis-Jean Couderc, Manish J. Butte, Alexandra F. Freeman, Émilie Catherinot, Claire Fieschi, Talal A. Chatila, Stuart G. Tangye, Holm H. Uhlig, Filomeen Haerynck, Jean-Laurent Casanova, Anne Puel

Vol. 217, No. 6 | 10.1084/jem.20191804 | March 24, 2020

The authors regret that in the original version of Table S1, the column for patient 12 was mistakenly duplicated in the column for patient 8.

The online Table S1 PDF has been corrected. The error appears only in PDFs downloaded before June 4, 2020.

